# Seven challenges for modelling indirect transmission: Vector-borne diseases, macroparasites and neglected tropical diseases

**DOI:** 10.1016/j.epidem.2014.08.007

**Published:** 2015-03

**Authors:** T. Déirdre Hollingsworth, Juliet R.C. Pulliam, Sebastian Funk, James E. Truscott, Valerie Isham, Alun L. Lloyd

**Affiliations:** a Mathematics Institute, University of Warwick, Coventry CV4 7AL, UK; b School of Life Sciences, University of Warwick, Coventry CV4 7AL, UK; c Department of Clinical Sciences, Liverpool School of Tropical Medicine, Pembroke Place, Liverpool L3 5QA, UK; d Department of Biology, University of Florida, Gainesville, FL 32611, USA; e Emerging Pathogens Institute, University of Florida, Gainesville, FL 32610, USA; f Fogarty International Center, National Institutes of Health, Bethesda, MD 20892, USA; g Centre for the Mathematical Modelling of Infectious Diseases, London School of Hygiene & Tropical Medicine, London WC1E 7HT, UK; h London Centre for Neglected Tropical Disease Research, Department of Infectious Disease Epidemiology, School of Public Health, Faculty of Medicine, Imperial College London, W2 1PG London, UK; i Department of Statistical Science, University College London, WC1E 6BT, UK; j Department of Mathematics and Biomathematics Graduate Program, North Carolina State University, NC 27695, USA

**Keywords:** EIP, extrinsic incubation period, EIR, entomological inoculation rate, FOI, force of infection, M&E, monitoring and evaluation, NTD, neglected tropical disease, VBD, vector-borne disease, VC, vectorial capacity, Vector-borne disease, Macroparasites, Neglected tropical diseases, Helminths, Vectors

## Abstract

Many of the challenges which face modellers of directly transmitted pathogens also arise when modelling the epidemiology of pathogens with indirect transmission – whether through environmental stages, vectors, intermediate hosts or multiple hosts. In particular, understanding the roles of different hosts, how to measure contact and infection patterns, heterogeneities in contact rates, and the dynamics close to elimination are all relevant challenges, regardless of the mode of transmission. However, there remain a number of challenges that are specific and unique to modelling vector-borne diseases and macroparasites. Moreover, many of the neglected tropical diseases which are currently targeted for control and elimination are vector-borne, macroparasitic, or both, and so this article includes challenges which will assist in accelerating the control of these high-burden diseases. Here, we discuss the challenges of indirect measures of infection in humans, whether through vectors or transmission life stages and in estimating the contribution of different host groups to transmission. We also discuss the issues of “evolution-proof” interventions against vector-borne disease.

## Introduction

The majority of core insights on the dynamics of infectious diseases are based on models of directly or sexually transmitted viruses or bacterial pathogens, as reflected in the other challenge papers in this issue. However, there are a huge number of pathogens which have multi-component transmission cycles, involving either vectors or complex pathogen life cycles. These pathogens present challenges in terms of the basic modelling structures and the extrapolation of insights from simpler systems to these complex systems and in more policy-related questions, as previously reviewed by other authors (Basáñez et al., 2012; Reiner et al., 2013; Smith et al., 2014).

Vector-borne diseases (VBDs), in which vectors, usually insects, take infection from one host to the next, are responsible for approximately 17% of the global infectious disease burden (World Health Organization, 2014). The most commonly modelled VBDs are malaria and dengue (Reiner et al., 2013), but many others cause a notable burden of disease in humans and other animals. There are a number of novel strategies being considered for VBDs, particularly for mosquito-borne infections, including biological controls (e.g. *Wolbachia*) and genetically modified vectors (McGraw and O’Neill, 2013; Sinkins and Gould, 2006), the success of which depend on our understanding of both the population dynamics of the vector and the transmission dynamics of the disease.

Macroparasites reproduce via infective stages outside the host, which generates different challenges for modelling their transmission. Despite a long history of macroparasite modelling (e.g. Anderson and May, 1991), the number of publications in this area is much lower than for directly transmitted pathogens, so there are many opportunities to apply recent advances in epidemiological modelling and statistical analyses in this area.

Neglected tropical diseases (NTDs) are a group of diseases that predominantly affect low-income populations in tropical countries. They include a wide range of infections, causative agents and routes of transmission, including macroparasites and VBDs, grouped for advocacy rather than epidemiological reasons. A number of NTDs lack well-defined models, and a diversity of approaches by multiple research groups is urgently needed (Kealey, 2010; The Lancet, 2014). Following several years of advocacy, these infections are now the subject of intense control efforts with many targeted for elimination over the next decades (WHO, 2012). As such, there are opportunities for novel mathematical modelling to inform the design of these programmes with immediate implementation and feedback, and a potentially large impact on human health.

Given the diverse nature of the infections covered here, we cannot hope to cover all the challenges in modelling for the future. We have therefore selected only 7 challenges within the groupings of (a) improvements in basic model structure, (b) contact processes and reservoirs of infection, (c) indirect measures of infection and (d) “evolution-proof” control. These challenges range from more technical modelling questions to clear biological or policy questions. They could arguably also have been grouped into those in which the structure of available models is not satisfactory or the modelling technique is not optimum (challenges 1, 4, 5 and 7) and those where the data have not been collected but the technical conditions to do so are present (challenges 2, 3 and 6).  ***Improvements in basic model structure***  

## 1. How can complex macroparasite processes best be modelled?

Macroparasitic infections (e.g. helminths and filarial nematodes) are characterized by relatively complex lifecycles and long time spans in the human host (from a few months to many years). Part of the parasite lifecycle is external to the host and there is no direct reproduction within the host, and therefore the burden of infection (e.g. number of helminths) can only increase through re-infection. The parasite load determines both transmission and morbidity of such infections. Importantly, this load can vary enormously between individuals, often well described by a highly overdispersed negative binomial distribution (Adler and Kretzschmar, 1992; Kretzschmar, 1993; Kretzschmar and Adler, 1993), an idea that goes back to Anderson and May (Anderson and May, 1978; May and Anderson, 1978). Thus, for macroparasites, a mathematical model needs to include the actual parasite load of each host, rather than simply tracking the total number of infectives. It may also be necessary to represent the various stages of the parasite lifecycle, in which there may be density-dependent effects, and to allow for parasite gender and mating. Furthermore, it is often desirable to incorporate immune responses to infection, and thus to include aspects of the infection history of each host. Multispecies infections are common, presenting additional complexity. While adding extra variables for each host is in principle straightforward, the increased complexity of additional state variables and nonlinearities inevitably means that exact results are difficult to obtain. Various approaches have been taken, including the use of hybrid models (Nasell, 1985) where stochastic variation of one or more variables is ignored. This can be a useful simplifying strategy when different aspects of the process are happening on very different timescales. For example, in a recent study of competition and coexistence of multispecies helminth infections (Bottomley et al., 2007), it was assumed that the free-living stage of the parasite is short relative to that of the adult worm and that their number is deterministic and in equilibrium.

Alternative, fully stochastic macroparasite models focus on particular aspects of the process, thus enabling analytic results. Often the aim is to eliminate some non-linear effects or to approximate them by linear ones. In early work (Tallis and Leyton, 1966, 1969), no interaction between the host and its parasites was allowed. Where appropriate, a useful simplification is to eliminate feedback in the infection cycle (Grenfell et al., 1995) or to assume there is direct infection of one host by another (Barbour and Kafetzaki, 1993). Analytic results can be obtained for models in which parasite-induced host mortality is the only source of nonlinearity and branching process approximations are a valuable tool (Herbert and Isham, 2000; Isham, 1995). Moment closure techniques can give helpful insight when the nonlinearities have suitably simple product forms (Grenfell et al., 1995).

Guidelines are needed on how best to approximate a complex system by a simpler one, clarifying those features that can reasonably be ignored while retaining those most responsible for determining its dynamics. There is a need for generic classes of fully stochastic and hybrid models to be identified that are applicable to groups of macroparasite infections.  ***Contact patterns and reservoirs of infection***  

## 2. Quantifying contributions of host and vector species for vector-borne infections with complex reservoirs

For any pathogen with multiple host species, the risk of cross-species transmission in a “target” host is determined by the spillover force of infection (spillover FOI). For zoonotic infections, where humans are the target host, this is the instantaneous hazard of animal-derived infection experienced by a susceptible human. For a directly transmitted zoonosis maintained in a single “reservoir” (non-human host) species, the spillover FOI can be calculated as the product of the prevalence in reservoir, the reservoir-human contact rate, and the probability of infection given contact (Lloyd-Smith et al., 2009). For zoonoses with complex reservoirs – i.e., those with multiple host species (and potentially multiple vector species) contributing to transmission – the spillover FOI is still a useful concept for quantifying human risk; however, an understanding of how transmission is maintained within and between the multiple reservoir species becomes essential for identifying both indirect and direct determinants of human risk and, therefore, for predicting the potential impact of proposed interventions.

Work on the ecology of tick-borne pathogens, such as *Borrelia burgdorferi* (the cause of Lyme disease) and Louping-ill virus, has emphasized that the ecology of the vector species – particularly the effects of different host species on vector abundance – must be taken into account to understand the contributions of specific wildlife species to pathogen maintenance, and that the role of a host species in determining risk to a target host may depend on the community composition of hosts and vectors (Gilbert et al., 2001; LoGiudice et al., 2003; Ostfeld and Keesing, 2000). For zoonoses with complex reservoirs, reduction of human risk via interventions targeted at animal hosts may be more effective, and will often be more cost-effective, than interventions targeted at humans; however, a formal framework for quantifying the contributions of hosts and vectors to pathogen invasion and persistence in specific settings will be needed to apply these approaches to the identification and evaluation of potential public health interventions. Identification of such interventions may be particularly important for VBDs occurring in resource-limited settings, where many of these diseases have the highest burden.

## 3. Understanding how contact patterns affect the dynamics of macroparasites

The contributions of different hosts to macroparasite transmission remains a key knowledge gap in our understanding of these pathogens. There are many mathematical and statistical tools for estimating and analysing transmission trees or infection processes for directly transmitted pathogens, but these have not yet been effectively adapted to macroparasitic modelling. Example research questions include:

*What processes generate the observed distribution of parasite load amongst hosts?* Macroparasitic infections are unevenly distributed, with some hosts having very high loads whereas others have very few (see discussion above). Some of this variation is maintained by ‘pre-disposition’ or the propensity of highly infected hosts to be quickly reinfected with high loads following treatment and re-exposure. For some macroparasites we also know that there are ‘wormy’ households, in which there are consistently higher parasite loads. Depending on the process which generates these aggregations, targeted control methods will have a greater or lesser effect. There is a need for a model structure which can unify these different observations through mechanistic, rather than statistical, formulations, in order to inform control programmes.

*How can we interpret the age distribution of loads to infer transmission dynamics?* Many, but by no means all, macroparasitic infections have their highest burden in children. Declining loads with age post-childhood is due to an undetermined combination of changing behaviour and developing immunity. Given this heterogeneity in loads and uncertainty in mechanism, and whilst still accounting for the household effects, are children or adults the major drivers of transmission? How does this affect the design of the most appropriate control strategies? Can we transfer insights from directly transmitted pathogens to macroparasites, or do the reinfection dynamics mean that targeted interventions are less efficient? These questions are similar to those posed for VBDs in a recent review of heterogeneities in transmission (Smith et al., 2014).

Within the context of directly transmitted infections, new data streams, including the availability of next generation sequencing and whole genome sequencing, have played an important role in improving inference of pathogen transmission patterns. Such data could similarly be used to improve inference of infection sources and transmission trees for macroparasites (Betson et al., 2013; Gower et al., 2013), and may be a useful source of information either for comparing mechanistic models or informing model construction by giving additional insight into the mechanisms that produce observed distributions of burden.  ***Indirect measures of infection and disease***  

## 4. Measuring vectors to estimate incidence and infection risk in humans

Vector-based surveillance programs are used as a risk assessment tool for many VBDs; however, the relationships between entomological measures of infection and human risk are non-linear, complicating the interpretation of such surveillance data. Models can be used to formalize and test assumptions that underlie such surveillance programs and to account for stochasticity and bias in the surveillance process itself, which may lead to improved interpretation of data and therefore more effective planning and intervention.

Entomological data often include trap counts, providing an indication of the relative temporal and/or spatial vector abundance, and prevalence of infection in the vector population (or related measures such as the minimum infection rate). Indeed, the product of vector density and the proportion of vectors that are infectious is closely related to several quantities that can be used to define risk of infection. For mosquito-borne infections, in particular, these measurements are often motivated by a desire to estimate vectorial capacity (VC—the expected number of hosts receiving bites from infectious mosquitoes per infected host per day (Smith and McKenzie, 2004)) or the entomological inoculation rate (EIR—the expected number of potentially infectious bites received per day by a susceptible host (Smith and McKenzie, 2004)). Sometimes more specific measurements (such as human landing catches, for malaria) are taken to directly quantify the human biting rate, which is a component of both VC and EIR.

Similarly, VBD models typically include the following assumptions regarding the relationships between quantities that define risk and entomological measurements:

• Vectorial capacity is proportional to the ratio of vector density to host density, resulting in invasion thresholds which are also are proportional to this ratio (Ross, 1905; Smith et al., 2012).
• Force of infection (FOI—the instantaneous hazard of infection experienced by a susceptible (host) individual), which is closely related to EIR, is proportional to the density of infectious vectors.

However, specific model formulations of these quantities often make additional assumptions that are not accounted for in the application of these formulae to data and the resulting interpretations of risk. One such assumption that is commonly overlooked (and is ubiquitously invalid, at least for mosquito populations) is that vector population density is constant. When vector density changes, prevalence of infection in vectors alone is insufficient to determine EIR, so the relationship between vector prevalence and risk breaks down, as does the commonly used approximation that the FOI is proportional to host prevalence (Dye and Williams, 1995). Nevertheless, risk assessments often use vector infection prevalence or related measures as the outcome of interest, as if this were a measure of risk–resulting in unaccounted for non-linear relationships between statistical assessments of “risk” and quantities of actual interest.

A more direct link between entomological measurements and quantities that define risk has been made for infections transmitted by some types of vectors – such as the use of density of infected nymphs, which is proportional to FOI, as the primary entomological indicator of risk in Lyme disease surveillance (Mather et al., 1996), however, even in these systems, modifying assumptions regarding homogenous biting, well-mixed encounters, temperature-driven changes in the external incubation period and vector life cycle, and other biological factors may be required to develop robust tools for risk assessment.

Rigorous, iterative frameworks should be sought to improve the links between the models used for risk assessment and the data to be interpreted (Koopman et al., 2014; Restif et al., 2012), and models should additionally account for the processes by which the entomological measurements themselves are generated. This area is ripe for leveraging recent statistical and computational developments that allow fitting of models to data via explicit treatment of latent variables involved in mechanistic processes and specification of observation models that can account for both stochasticity and known biases in the mechanisms by which data are generated (Bretó et al., 2009).

## 5. Develop robust models for interpreting indirect measures of macroparasitic infection

Relating models to data is a general epidemiological challenge (Lessler et al., 2015). However, for almost all macroparasitic infections, our most commonly used measures of the intensity of infection are indirect. This is particularly true of helminth infections, where we very rarely observe the adult worm burden, but rather transmission stages, such as microfilariae or egg output. Where worm burdens can be measured, we know that there are complex, non-linear, density-dependent relationships between these indirect measures and the underlying worm burden. For example, there is a density dependent relationship between worm burden and egg output for soil-transmitted helminths, which is further complicated by variability in egg output from one sample to the next and from one day to the next. As discussed above, models of macroparasitic diseases are formulated in terms of the dynamics of the various stages of the parasite's development. In order to fit models to such data, it is necessary to infer information about the distribution of parasites from measurements of egg output and prevalence. As yet, little attention has been paid to this process.

A key-modelling question is: what models should be used to capture the relationship between parasites and egg output (or other indirect measurements)? This includes the dependence of egg output on parasite density and the mode of sexual reproduction of the parasite as well as the effectiveness of the measurement protocol used to count eggs.

Equally, an understanding of the nature and sources of variance in egg production and measurement are essential to any statistical inference of underlying worm distributions. Given that variances are characteristically large, it will be necessary to develop statistical approaches that can integrate many different sources of relatively ‘weak’ data to arrive at the strongest possible inference for underlying parasite populations.

The development of probabilistic models, as described above, could have implications for study design and monitoring and evaluation (M&E). Given a particular statistic of interest (e.g. mean parasite burden in schoolchildren), it would be possible to optimize study design and the process of M&E to maximize the information recovered from the target population as a function of the cost.

## 6. Estimating burden for NTDs

NTDs are by definition underobserved, often because of limited access to health care or lack of diagnostic or recording capabilities. This is compounded by the difficulties due to indirect measures of infection (see challenges above). In many settings, cases are found through active detection campaigns, but otherwise remain unrecorded. This can lead to reported case series that do not reflect the true dynamics: more investigation leads to better detection and thus more reported cases, while a reduction of reported cases can be a consequence of either effective control or a breakdown in surveillance. Accurate burden estimates, however, are crucial to predict the likely impact of, and resources needed for, control efforts. The challenge here is to develop models that can combine patchy data to fill the gaps and produce reliable burden estimates in the absence of routine surveillance. Using state-of-the-art methods for model fitting and inference (e.g. Monte Carlo-based methods (Andrieu et al., 2010; Liu and West, 2001; O’Neill, 2010)), transmission dynamics can be combined with a variety of data taken at different time points (e.g. limited routine surveillance combined with active case detection) to estimate the most likely underlying burden. Moreover, these could yield estimates for disability/quality-adjusted life years lost, an important currency in the economics of disease control. Combined with age structure or spatial information, such analyses could make a valuable contribution for targeting control efforts within the WHO roadmap (WHO, 2012).  ***Evolution-proof control in the presence of large-scale interventions***  

## 7. Evolution-proof control of vectors

VBD lifecycles present multiple targets for control efforts, e.g. reducing vector density by insecticides or shortening the duration of human infectiousness using drug treatments. Deployment of an effective control measure against an agent inevitably imposes a strong selective pressure for evolutionary escape from that measure. Vector-borne infections are no exception: control efforts against malaria, as an example, are threatened by evolution of resistance to insecticides (Hemingway and Ranson, 2000) and antimalarial drugs. Behavioural evolution of vectors, for instance shifting from indoor to outdoor biting in response to control measures such as indoor residual spraying or insecticide-laced bed nets is an additional concern (Gatton et al., 2013).

Understanding the evolutionary implications of control measures is, therefore, a key task. Much of the work that has been undertaken has direct analogies to questions asked for directly transmitted infections, such as whether multiple forms of a control (e.g. insecticides or drug treatments) should be used in combination or in a cyclic fashion. There are, however, some important differences: the observation that the latent period of infection within the vector–the extrinsic incubation period (EIP) – is often a substantial fraction of the average adult female lifespan raises ways to lessen the evolutionary impact of control, dubbed “evolution-proof control” (Read et al., 2009).

It has long been realized that the lengthy EIP and the need for a female mosquito to feed twice to first acquire and then transmit the pathogen means that old females are responsible for the majority of transmission events and that even modest reductions in mosquito lifespan could result in significant reductions in transmission (Macdonald, 1956). Consequently, “late-acting” control measures, such as late-acting insecticides (Read et al., 2009) or life-shortening *Wolbachia* bacteria (McGraw and O’Neill, 2013), could effectively control transmission while imposing much reduced selection pressure on the mosquito population, acting after the majority of a mosquito's offspring have been produced. Evolution might still have the last word here as there would be pressure for the pathogen to shorten its EIP.

Modelling challenges here include exploring the impacts of combinations of control measures and whether there are epidemiological and/or evolutionary synergies to using multiple control measures, even if some are somewhat ineffective individually. Consideration of a wide range of control options – including release of sterile mosquitoes, paratransgenesis, and late-acting or life-shortening mosquito-control techniques in combination with more traditional measures – and their combined evolutionary implications could yield substantial insights that would be useful reducing burden and eventual elimination (Macdonald, 1956; McGraw and O’Neill, 2013; Read et al., 2009).

## Summary

This article covers a huge range of infections for which we have an increasing amount of experimental, epidemiological, entomological, ecological, clinical and monitoring and evaluation data. Many of the issue of how to control and even eliminate these infections will be addressing challenges in other articles in this issue (Klepac et al., 2015; Metcalf et al., 2015), but they pose unique challenges either due to their complexity (through vector-borne transmission or their macroparasitic life cycles), or due to a limited amount of biological, ecological or epidemiological data. They are potentially the infections where most novel epidemiological insights will be made over the coming decades.
